# Cognitive trajectories preluding the onset of different dementia entities: a descriptive longitudinal study using the NACC database

**DOI:** 10.1007/s40520-024-02769-9

**Published:** 2024-05-23

**Authors:** Ioannis Liampas, Nefeli Dimitriou, Vasileios Siokas, Lambros Messinis, Grigorios Nasios, Efthimios Dardiotis

**Affiliations:** 1 Department of Neurology, School of Medicine, University Hospital of Larissa, University of Thessaly, Mezourlo Hill, Larissa, 41100 Greece; 2 Department of Speech and Language Therapy, School of Health Sciences, University of Ioannina, Ioannina, 45500 Greece; 3 Laboratory of Cognitive Neuroscience, School of Psychology, Aristotle University of Thessaloniki, Thessaloniki, 54124 Greece

**Keywords:** Alzheimer’s disease, Lewy body dementia, Vascular dementia, Frontotemporal Lobal degeneration

## Abstract

**Objective:**

To describe the 10-year preclinical cognitive trajectories of older, non-demented individuals towards the onset of the four most prevalent types of dementia, i.e., Alzheimer’s disease(AD), Lewy body(LBD), vascular(VD) and frontotemporal dementia(FTD).

**Methods:**

Our analysis focused on data from older (≥ 60years) NACC (National Alzheimer’s Coordinating Center) participants. Four distinct presymptomatic dementia groups (AD-LBD-VD-FTD) and a comparison group of cognitively unimpaired(CU) participants were formed. Comprehensive cognitive assessments involving verbal episodic memory, semantic verbal fluency, confrontation naming, mental processing speed – attention and executive function – cognitive flexibility were conducted at baseline and on an approximately yearly basis. Descriptive analyses (adjusted general linear models) were performed to determine and compare the yearly cognitive scores of each group throughout the follow-up. Exploratory analyses were conducted to estimate the rates of cognitive decline.

**Results:**

There were 3343 participants who developed AD, 247 LBD, 108 FTD, 155 VD and 3398 composed the CU group. Participants with AD performed worse on episodic memory than those with VD and LBD for about 3 to 4 years prior to dementia onset (the FTD group documented an intermediate course). Presymptomatic verbal fluency and confrontation naming trajectories differentiated quite well between the FTD group and the remaining dementia entities. Participants with incident LBD and VD performed worse than those with AD on executive functions and mental processing speed-attention since about 5 years prior to the onset of dementia, and worse than those with FTD more proximally to the diagnosis of the disorder.

**Conclusions:**

Heterogeneous cognitive trajectories characterize the presymptomatic courses of the most prevalent dementia entities.

**Supplementary Information:**

The online version contains supplementary material available at 10.1007/s40520-024-02769-9.

## Introduction

Cognitive impairment precedes dementia onset by many years. Preclinical cognitive trajectories have been mainly studied in individuals with incident Alzheimer’s disease dementia (AD). Published evidence suggests that cognitive changes tend to appear more than a decade earlier than the formal identification of AD [1, 2], while an accelerated course of decline is observed 3 to 5 years prior to the onset of the disorder (depending on the specific domain of cognition) [3–5]. Episodic memory is consistently reported to be the first and most conspicuously affected domain of cognition throughout the presymptomatic course of AD [1, 2, 6]. On the other hand, there are only scant data on the preclinical cognitive trajectories of other dementia types.

Older adults with Lewy body pathology have been found to outperform those with AD in terms of episodic memory while exhibiting worse attention, executive function and visuospatial scores over the presymptomatic course of dementia [7, 8]. Similarly, those with incident vascular dementia (VD) have been reported to outperform patients with future AD on episodic memory tasks prior to the onset of dementia and to perform worse on executive function, attention and visuoperceptual assessments [9–11]. Neither Lewy body dementia (LBD) nor VD exhibit substantial language differences compared to AD during the preclinical course. Of note, there is even more scarce evidence on the preclinical trajectories of individuals converting to frontotemporal dementia (FTD), without any direct comparison with those progressing to AD [12, 13].

Along with AD - LBD, VD and FTD (including mixed pathologies) constitute the most common types of dementia and compose the principal differential diagnoses of dementia cases [14]. A vast body of the published literature has focused on the precise and accurate identification of the imminent onset of these entities in older non-demented adults [15, 16]. Together with motor manifestations and neuropsychiatric symptoms, cognitive measures are universally considered an integral part of the armamentarium of clinical predictors of cognitive impairment and incident dementia [17–20]. Considering the crucial prognostic contributions of preclinical cognitive assessments, we decided to undertake the current study in order to describe the 10-year preclinical cognitive trajectories of older, non-demented individuals towards the onset of the four most prevalent types of dementia, i.e., AD, LBD, VD and FTD. For this purpose we capitalized on data from the Uniform Data Set (UDS) [21, 22]. We aspired that our study would provide both clinicians and researchers with an additional screening tool in the challenging task of differentiating the presymptomatic courses of the main dementia entities.

## Methods

### Population and settings

UDS is stewarded by the National Alzheimer’s Coordinating Center (NACC) since 2005. It constitutes a central repository of longitudinally collected data from multiple Alzheimer’s Disease Research Centers (ADRCs) across the United States. The key features of the database have been described elsewhere [23–25]. In short, UDS enrols clinician-, self- or family-referred volunteers, as well as actively recruited individuals with a cognitive status ranging from normal cognition to full-blown dementia, in accord with each ADRC’s discrete protocol. Participants are comprehensively evaluated according to a standardized approach, on an approximately yearly basis. Written, informed consent is obtained from all participants or surrogates before participation. The Institutional Review Boards of each ADRC monitors all procedures in accordance with the ethical standards of the 1964 Declaration of Helsinki and its later amendments.

### Participant selection and diagnostic procedures

This analysis focused on data from older (≥ 60 years at baseline) NACC participants, enrolled between September 2005 (year UDS was established) and December 2022 (data freeze), from a total of 46 ADRCs. Participants without dementia at baseline were considered for eligibility. Four distinct preclinical dementia groups were formed, including those who developed AD, LBD, VD or FTD at follow-up, respectively. The remaining participants that developed dementias primarily associated with alternative neurodegenerative or non-neurodegenerative causes were excluded: e.g., Huntington’s disease, traumatic brain injury, normal pressure hydrocephalus, central nervous system neoplasm, psychiatric disorder, alcohol, or other substance abuse, progressive supranuclear palsy, corticobasal degeneration, and so on. A group of ‘‘healthy’’ comparators was also shaped. The latter featured those that remained CU throughout the follow-up and had a minimum monitoring of 6 visits (to balance between including truly CU individuals with many serial normal evaluations and maintaining sufficient power).

In the context of the UDS, the diagnoses of CU, MCI and dementia are established by either the examining physician or (in the vast majority) by an expert-consensus panel, in accord with the distinct protocol of each ADRC, using standard clinical criteria [26–31]. Cognitively impaired participants who do not clearly fit into the categories of MCI or dementia are identified as cognitively impaired – not MCI. Participants are classified as CU in the absence of cognitive impairment (dementia, MCI, or cognitive impairment not MCI). Diagnostic biomarkers are only available in a minority of cases. The diagnostic classification of the participants (stage of cognitive impairment and main underlying cause) is updated during each re-assessment and incongruity among serial clinical diagnoses is occasionally documented (e.g., the presumed primary cause of cognitive impairment might alternate between AD and VD, or even among more than two dementia entities, for instance AD-VD-LBD, and so on).

Considering the challenging differential diagnosis of early AD, LBD, VD and FTD, to refine the participant selection process and at the same time maintain a fair statistical power, the following algorithm was devised for the current analysis: (1) in cases with four or more diagnostic assessments, the most persistent cognitive diagnosis was utilized, according to the following criterion: only those with a consistent diagnosis in ≥ 70% of their visits were classified as having the corresponding dementia entity, e.g., cognitive diagnoses of AD in 3 out of 4 visits, or 4 out of 5, or 5 out of 6, and so on. (2) Those with four or more diagnostic assessments not fulfilling the aforementioned prerequisite were excluded from every analysis. (3) Those with two or three diagnostic assessments without complete concordance were also excluded from every analysis. Those with one diagnostic assessment (no post-conversion assessment) were assigned to the diagnosis determined at that single visit.

### Measurement of cognitive performance

All three UDS versions focused on the cognitive domains of verbal episodic memory, semantic verbal fluency, confrontation naming, mental processing speed – attention and executive function – cognitive flexibility [25]. In the first two versions of the UDS, verbal episodic memory was assessed on the Logical Memory Test - Story A (LMT-SA) from the Wechsler Memory Scale—Revised (WMS-R) [32], confrontation naming according to the 30-item version of the Boston Naming Test (BNT-30) [33], semantic verbal fluency on the total word production summing the Animal and Vegetable Fluency Tasks [34], mental processing speed – attention on the Trail Making Test—Part A (TMT-A) and executive function – cognitive flexibility on the Trail Making Test—Part B (TMT-B) [35]. The administration and scoring of these tests has been detailed elsewhere [25]. In the third most recent UDS version, semantic verbal fluency, mental processing speed – attention and executive function were evaluated on the same neuropsychological tasks, whereas verbal episodic memory and confrontation naming were assessed on the Craft Story 21 (CS-21) [36] and Multilingual Naming Test (MINT) [37], respectively. The administration and scoring of these tests has been detailed elsewhere [38]. To limit the amount of missing data, CS-21 and MINT scores were converted to LMT-SA and BNT-30 scores correspondingly, according to the detailed conversion tables provided by the NACC crosswalk study [39].

### Outcome measures and statistical analysis

The primary purpose of the current analysis was to determine the preclinical longitudinal cognitive trajectories towards the onset of AD, LBD, VD and FTD. The main trunk of the current analysis was based on a descriptive approach. Descriptive statistics were adjusted for age, sex, race, education and time (in years) prior to the former identification of dementia [40, 41]. Age at baseline and education in years of formal schooling were treated as scale variables. Sex and race (Caucasian, African American, American Indian, or Alaskan native, native Hawaiian or Pacific islander, Asian and other) were treated as categorical variables. Adjusted mean cognitive scores (episodic memory, verbal fluency, naming, processing speed – attention and executive function) and precision estimates were determined yearly, throughout a 10-year preclinical follow-up (year − 10, -9, …, -1, 0), via univariate general linear models (GLMs). In other words, separate adjusted GLMs were performed per cognitive score, per yearly assessment (i.e., a total of 11 GLMs were performed per cognitive assessment). In case a participant had more than one assessment at ± 6 months around a given year, we capitalized on the assessment closer to the designated year and excluded any additional measurements to eliminate intercorrelation between/among repeated assessments. The minimum of 10 individuals per assessment, per dementia group, was prespecified for inclusion of each participant group in the respective GLM.

As a secondary outcome, we compared the rates of cognitive decline among the different participant groups using generalised estimating equations (GEE) analyses. GEE accounts for the potential correlation of repeated measurements in the same individual. We treated each participant’s serial evaluations as a cluster. Conventionally, exchangeable covariance matrices were used as working correlation structures. Consecutive GEE models were explored using the individual domain cognitive measurements (episodic memory, verbal fluency, naming, attention, processing speed – attention and executive function – cognitive flexibility) as the dependent scale variables. All models were adjusted for the same set covariates as above. For each cognitive outcome, two independent GEE models were performed, one for the last four years prior to dementia onset (adequate data for the FTD group were only available for this short timespan) and one for the complete 10-year follow-up (participants with FTD at follow-up were not included in this analysis).

Statistical analyses were performed using the IBM SPSS Statistics Software Version 26 (Chicago, IL, USA). Despite performing multiple GLMs (per cognitive score per year), given the exploratory nature of our analyses, the conventional threshold of α = 0.05 was implemented for the revelation of statistical significance. However, each separate GLM (between group comparisons) was adjusted according to the Bonferroni correction.

## Results

**Fig. 1:**
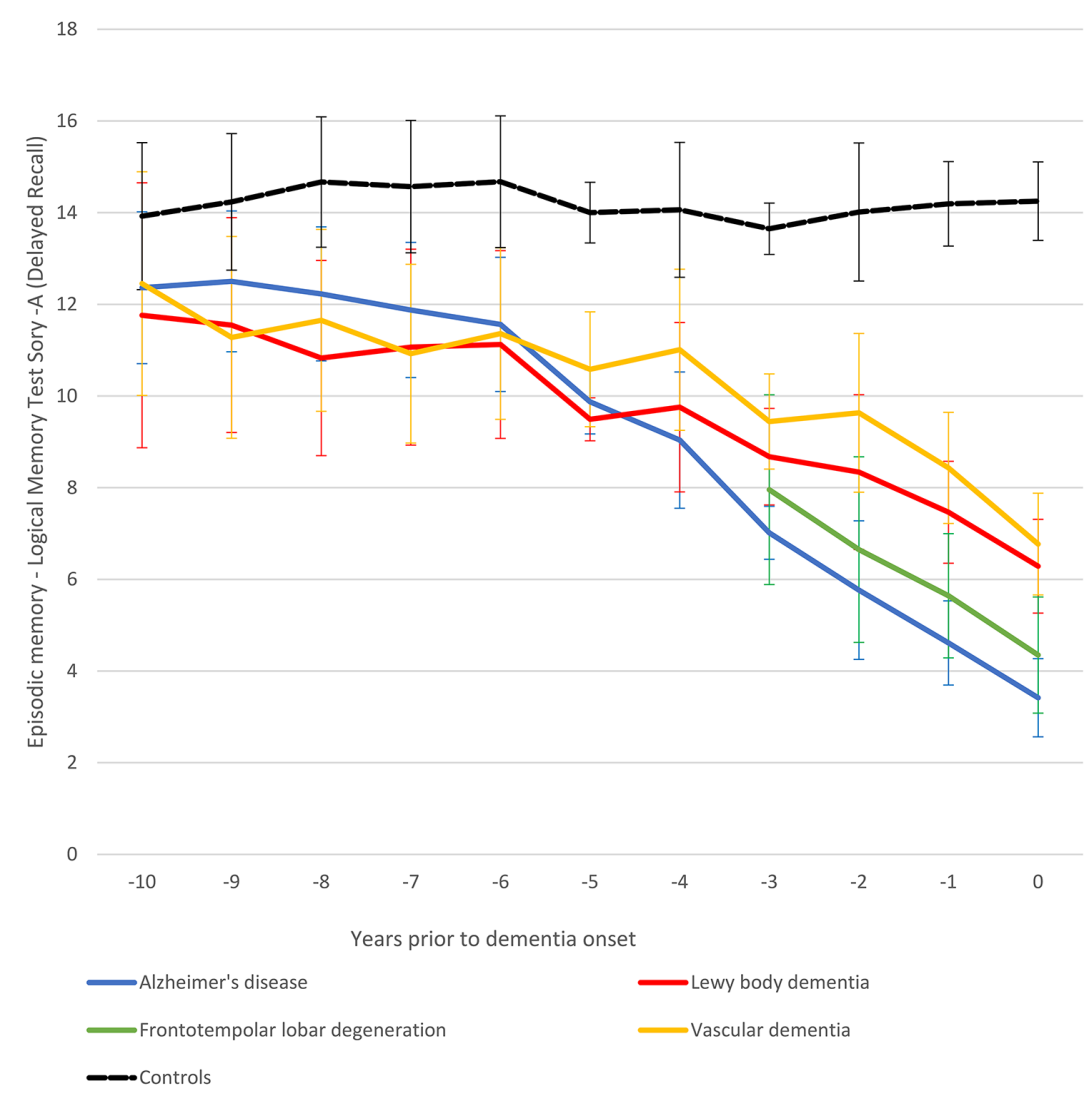
Episodic memory trajectories towards dementia onset. Average performances and precisions estimates (95% confidence intervals) are presented

**Fig. 2:**
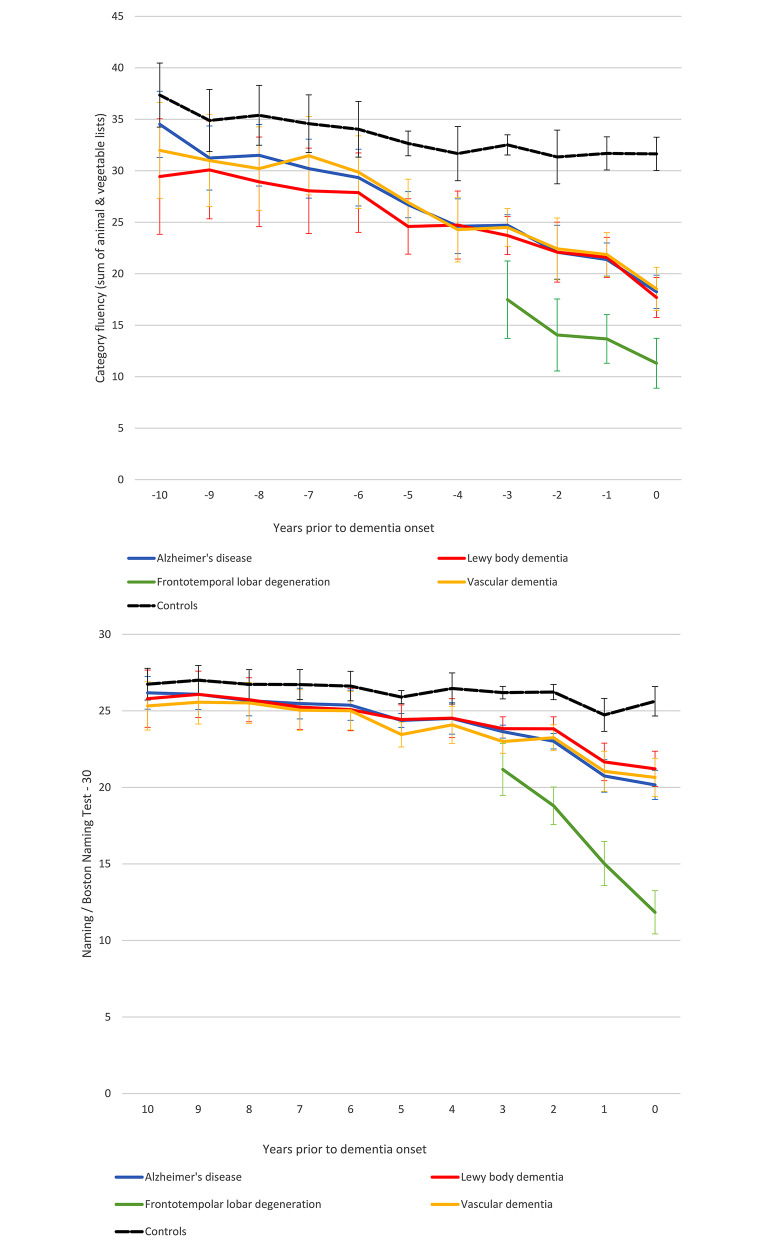
**(a)** Verbal fluency trajectories towards dementia onset; **(b)** Naming trajectories towards dementia onset. Average performances and precisions estimates (95% confidence intervals) are presented

**Fig. 3:**
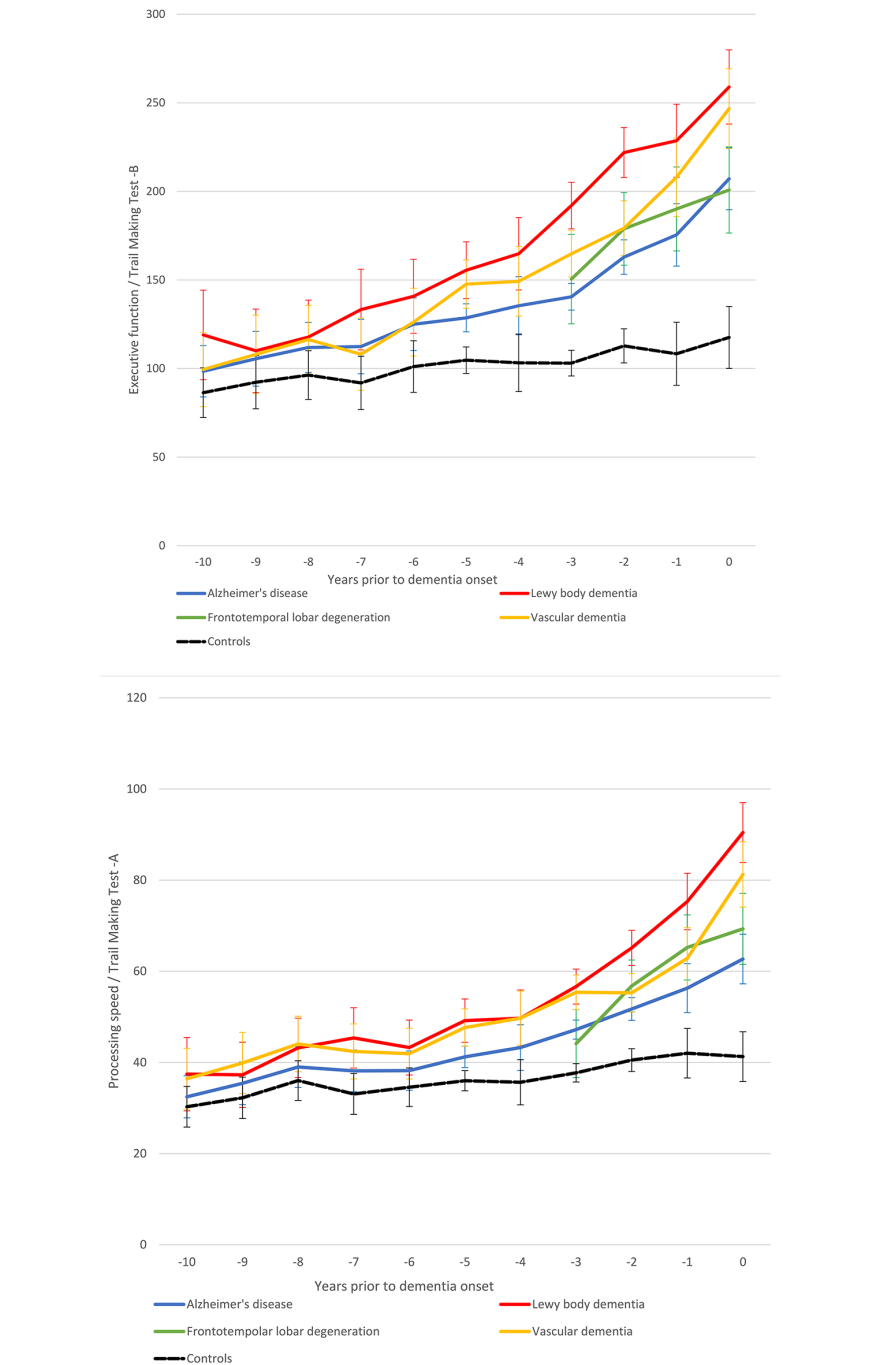
**(a)** Executive function trajectories towards dementia onset; **(b)** Processing speed - attention trajectories towards dementia onset. Average performances and precisions estimates (95% confidence intervals) are presented

From the 47,165 NACC participants, 7251 were included in the current analysis (Supplementary Fig. 1). Among them, 3343 progressed to AD, 247 converted to LBD, 108 developed FTD, 155 were diagnosed with VD and 3398 remained CU throughout the follow-up. Patient demographics are illustrated at Supplementary Table 1. In brief, our older, well-educated sample consisted predominantly of women of Caucasian ancestry. However, female predominance was observed in the LBD group, while men and women were equally represented in the FTD group. The longitudinal cognitive trajectories by dementia diagnosis at follow-up are illustrated in Figs. 1, 2 and 3. Exact numbers of participants analysed as well as detailed descriptive statistics on our sample’s cognitive performance throughout the 10-year follow-up are provided in the Supplementary Tables 2–7. Due to limited data availability, only 3-year trajectories were constructed for the FTD group (the prerequisite of 10 participants per yearly assessment, per dementia group was not fulfilled for the FTD group over the earlier preclinical course).

### Cognitive trajectories based on the descriptive approach – results from the GLMs

Episodic memory trajectories differed between individuals with and without incident dementia practically throughout the 10-year follow-up. Among those with future dementia, episodic memory trajectories diversified more proximally to the formal dementia diagnosis (Fig. 1). Participants with AD presented lower episodic memory scores compared to those with VD and LBD for about 4 and 3 years earlier from the formal identification of dementia, respectively. Moreover, individuals with FTD performed worse than those with VD and LBD for 2 and 1 years prior to dementia onset, correspondingly. At the same time, the VD and LBD groups composed an intermediate episodic memory class that performed significantly worse than CU individuals from as early as 9 years prior to dementia onset, but clearly outperformed those with future AD-FTD more proximally to the development of dementia. Detailed descriptives and between group differences are in Supplementary Table 3.

Semantic verbal fluency and confrontation naming trajectories (belonging to the broad domain of language) differentiated between the FTD group and the remaining dementia entities quite well (Fig. 2). In specific, those with incident FTD performed worse than those converting to other dementia entities in both language measures, essentially throughout the 3-year follow-up. On the other hand, although semantic verbal fluency and confrontation naming trajectories of individuals with future AD, LBD and VD diverged from those with intact cognition from early on, no particular differences were established among the 3 groups over the 10-year monitoring. Detailed descriptives and between group differences are presented in Supplementary Tables 4–5.

Executive function and mental processing speed – attention trajectories appeared to abide by a similar pattern of decline (Fig. 3). Participants with incident LBD and VD performed worse than those with AD on executive function since about 5 years prior to the onset of dementia. At the same time, the FTD group outcompeted the LBD group throughout the 3-year follow-up and outperformed the VD group at the time of dementia onset. As for mental processing speed – attention, again those with future LBD and VD documented lower scores compared to the AD and FTD groups over the same preclinical periods of time. Of note, near the formal identification of dementia both measures diversified between LBD and VD participants, with the latter outperforming the former. Detailed descriptives and between group differences are in Supplementary Tables 6–7.

**Fig. 4:**
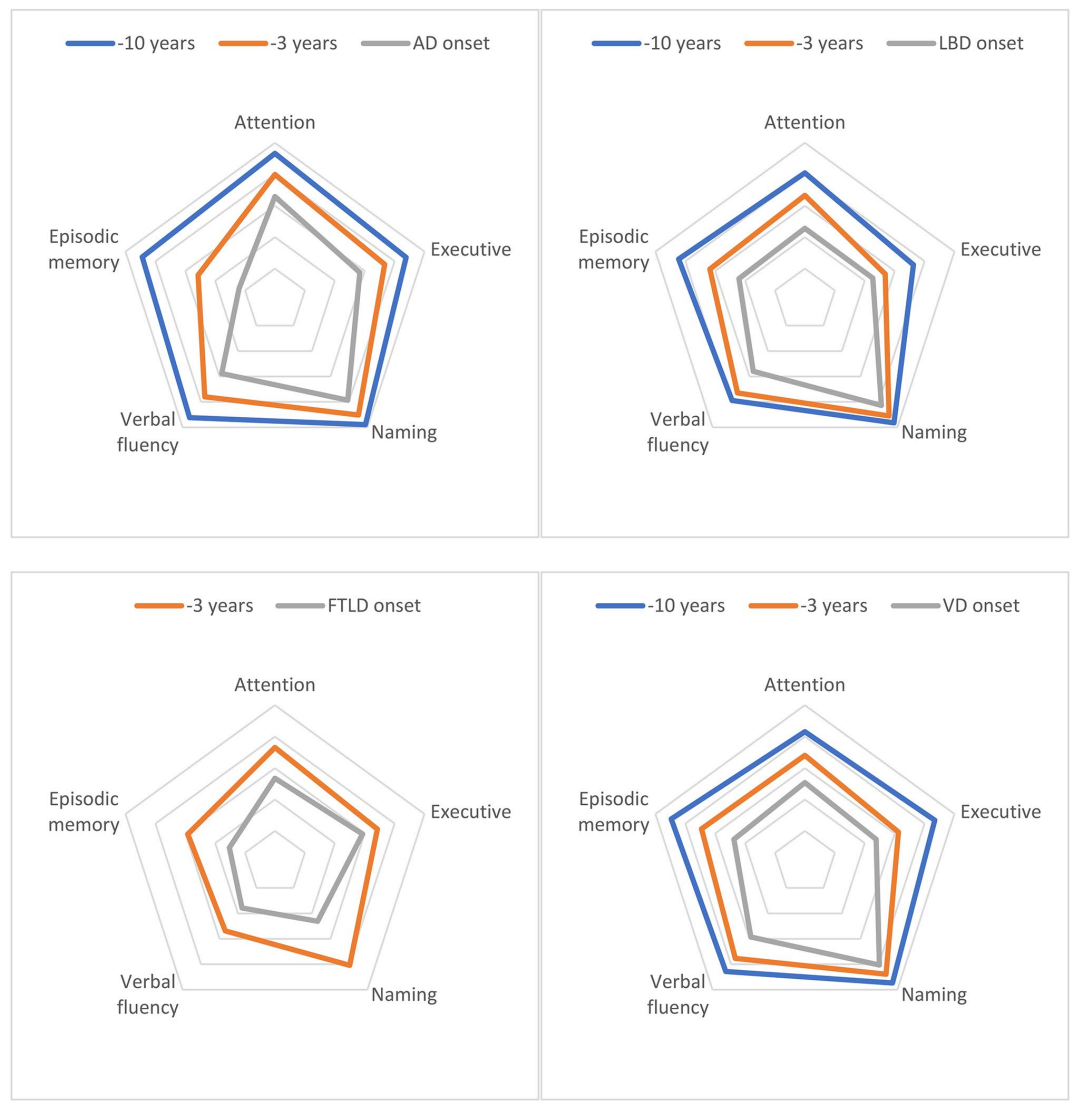
Radar plots illustrating relative (to healthy controls) cognitive deficits per dementia type, 10 years prior to dementia onset, 3 years prior to dementia onset and at the time of the formal diagnosis. The external regular pentagon represents the performance of those without cognitive impairment throughout the follow-up. Centripetally and respectively, the remaining pentagons correspond to 80%, 60%, 40% and 20% of the performance of the cognitively unimpaired sample. AD: Alzheimer’s disease dementia; LBD: Lewy body dementia; VD: vascular dementia; FTLD: frontotemporal lobal degeneration dementia

Figure 4 and Supplementary Fig. 2 illustrate the relative (to healthy controls) cognitive deficits of the four most common dementia types at three key preclinical points: 10 years prior to dementia onset, 3 years prior to dementia onset and at the time of the formal diagnosis. Regarding AD, a much more prominent episodic memory dysfunction was observed compared to the remaining cognitive domains. On the other hand, a rather homogeneous decline in executive function, attention, and episodic memory (verbal fluency was less conspicuously affected) with relatively intact naming was found for LBD and VD. Finally, language components along with episodic memory were steeply affected by FTD.

### Rates of cognitive decline – results from the GEEs

As indicated from the descriptives of our sample, the rates of episodic memory decline were more prominent among those with incident AD and FTD (∼ 1.2 fewer words recalled per annum, over the last 4 years prior to dementia onset compared to the CU group) (Supplementary Table 8). Episodic memory scores diminished by ∼ 1 word yearly in individuals with future VD and by ∼ 0.8 words yearly in those with future LBD over the 4-year preclinical course leading to dementia onset, in comparison with CU older adults. Of note, the 10-year rates of episodic memory decline were almost double in the AD (early β= -0.87) compared to the LBD (early β= -0.44) and VD groups (early β= -0.48).

Verbal fluency (∼ 2.5 fewer responses per annum vs. CU) and confrontation naming (∼ 2.7 fewer responses per annum vs. CU) declined in a conspicuous fashion among those with future FTD over the 4-year preclinical follow-up before dementia. Less abrupt trajectories were documented among those with incident AD, LBD, and VD. On the other hand, executive function – cognitive flexibility, and processing speed – attention decline rates were markedly elevated among participants with LBD (β = 21.5 and β = 10.4, respectively) and VD (β = 23.9 and β = 7.5, respectively) over the last 4 years prior to dementia onset. Of note, in both groups and especially LBD steep rates of executive function (β = 16.0 for LBD and β = 12.5 for VD) and processing speed decline (β = 5.6 for LBD and β = 3.2 for VD) were documented throughout the 10-year follow-up.

## Discussion

The present study explored whether the preclinical cognitive courses of individuals with incident dementia diversify among individuals converting to AD, FTD, LBD and VD, up to 10 years before the formal clinical diagnosis of dementia. Our analysis revealed that verbal episodic memory, semantic verbal fluency, confrontation naming, mental processing speed-attention and executive function - cognitive flexibility evolved differently throughout the preclinical phase of these four most common types of dementia. More specifically, individuals with future AD performed worse on episodic memory tasks compared to those who developed VD and LBD for about 4 and 3 years before the ascertainment of the disorder, respectively. Participants with FTD performed worse than those with VD and LBD for 2 and 1 years prior to dementia onset, correspondingly. Verbal fluency and naming scores were conspicuously worse throughout the 3-year preclinical follow-up in those who developed FTD. Executive function and processing speed – attention trajectories differentiated between those with future LBD and VD and those with incident AD about 5 years before the formal identification of the disease Finally, individuals who progressed to LBD and VD performed worse than those who converted to FTD more proximally to the formal identification of the disorder.

These preclinical findings appear to align with the common patterns of cognitive impairment observed during the symptomatic course of dementia. In more detail, episodic memory impairment is long-considered the hallmark of AD pathology; however, other major dementia syndromes affect episodic memory performance sufficiently to lead to diagnostic challenges [42]. On the other hand, language impairment – confrontation naming or category fluency impairment in specific - are more pronounced among those with FTD [43, 44]. Regardless, both naming and semantic fluency deficits are apparent in individuals with AD and may complicate the identification of the correct dementia entity [45]. Of note, letter fluency might enhance the diagnostic accuracy of category fluency tasks, since it is often comparably affected in patients with FTD and relatively preserved in individuals with AD [46]. Finally, executive function and attention – processing speed impairments are disproportionately affected by LBD and VD [8]. Although dysexecutive AD and FTD appear to share these cognitive deficits, the comprehensive assessment of cognition usually reveals concomitant episodic memory and language deficits, respectively, that help differentiate these entities.

Our findings are also in accordance with published evidence on the pre-diagnostic AD trajectories, Previous research found that subtle cognitive changes may often start as early as 15 years before the formal diagnosis of AD [1]. The majority of those who eventually develop AD, however, usually exhibit accentuated rates of cognitive decline over the last 3–8 years prior to the formal identification of the disorder [47, 48]. Episodic memory impairment manifests earlier, whereas language, visuo-perceptual skills, executive function and attention ensue about 3–5 years before onset [3, 4, 9, 49]. Episodic memory constantly exhibits the worst relative trajectory throughout the pre-diagnostic phase (the most prominent deficits compared to the other functions) [6]. More variation exists with respect to the remaining pre-diagnostic cognitive trajectories.

Increasing emphasis is placed on the preclinical identification of individuals that will ultimately develop dementia The recognition of this particular subgroup offers substantial clinical advantages in terms of better management and minimization of iatrogenic complications [50]. At the same time, ongoing research focuses on the presymptomatic application of preventive interventions with an aim to delay the onset of -prevent if possible- dementia [51]. Of note, apart from affected individuals, family members and caregivers could as well benefit from the early identification of incident dementia, by preparing for the upcoming demands and seeking appropriate support, beforehand [52].

Given the numerous advantages of the presymptomatic diagnosis, the elaboration of various tools and procedures with valuable prognostic accuracy is crucial. Such tools vary from simple and inexpensive clinical examinations such as assessment of neuropsychological measures [53], motor functions (e.g., gait performance) [54], neuropsychiatric manifestations (e.g., psychotic symptoms, affective disorders, lability symptoms and so on) [55] or sleep parameters [8], to more sophisticated, costly and occasionally interventional evaluations, namely imaging (from simple structural magnetic resonance imaging and single-photon emission computed tomography, to dopamine transporter scan and positron emission tomography) and cerebrospinal fluid biomarkers (β-amyloid, tau and phospho-tau; and potentially α-synuclein) [56, 57]. Of note, novel blood-based biomarkers of AD are less invasive and more cost effective, more appropriate for repeat testing and monitoring, offering overall the chance for larger-scale applicability [58]. Amyloid-β, tau, phospho-tau, GFAP (glial fibrillary acidic protein) and Nf-L (neurofilament light) are the most promising; however, considerable challenges have yet to be tackled in order to ascertain the diagnostic value of these indices [59, 60]. Of note, there are even more elaborate detection strategies, that capitalize on a constellation of the aforementioned factors, which in combination tend to capture a larger proportion of the risk-variation and predict the onset of future dementia with greater accuracy [61]. Considering the impractical and often time-wasting nature of entirely clinical approaches, it is probably more optimal to capitalize on clinical evaluations as an initial ‘sieve’ to distinguish those at high-risk of incident dementia and followingly turn to more sophisticated laboratory exams to establish an accurate early - ‘‘preclinical’’ identification of the underlying major neurocognitive entity.

The aim of the current report was to enhance the prognostic properties of preclinical neuropsychological evaluations via the exploration of the preclinical trajectories of the four most common type of dementia. Although the presymptomatic trajectories of AD have been a matter of extensive research, less was known about the preclinical cognitive course of DLB, VD and FTD. Future research ought to confirm and combine our findings in the search of more sensitive and precise predictive models that will serve preclinical detection purposes of individuals without dementia at high-risk of converting to AD, DLB, VD and FTD.

### Strengths and limitations

The main strengths of our study are the large sample size and the long follow-up period. To reduce the miscategorization of dementia cases, an algorithm accounting for incongruent serial diagnoses throughout the follow-up was devised. A group of CU comparators with at least 6 normal serial assessments was formed.

This analysis has several limitations, as well. First, the diagnosis of dementia was established by either the examining physician or an expert-consensus team, based on comprehensive neurological and neuropsychological evaluations (imaging and biological biomarkers were not uniformly available). Although, the exhaustive assessments of the UDS improve the accurate diagnostic characterization of the participants, the presence of misclassification bias cannot be ruled out, especially for cases of mixed dementia. Second, the prevalence of LBD, VD and FTD were expectedly low because of the AD-focused nature of the NACC dataset, underpowering at least some of our analyses. This is reflected in the large precision estimates of our findings and may have obscured several non-trivial associations. Third, in view of the small number of individuals with incident FTD, the behavioral phenotype was clustered together with language variants; of note, previous studies suggest that the relative proportion of FTD subtypes in the NACC UDS is ∼ 70% for behavioral variant FTD and ∼ 70% for primary progressive aphasias (∼ 15% for non-fluent variant FTD and ∼ 15% for semantic variant FTD) [62]. Given the heterogeneous cognitive profiles of these entities, lumping them together may have to some extent distorted their potentially disparate prediagnostic cognitive trajectories. In addition, the UDS study population is not a statistically based sample of the US population; participants tend to be highly educated and there is over-representation of Caucasians and women. Therefore, readers should be cautious with the generalizability of our findings. Moreover, although we adjusted all analyses for several factors and covariates, our findings may have been driven by residual confounding (it would not be possible to capture the effect of every potential confounder) or the non-trivial proportion of missing data [63, 64]. Finally, another limitation of this study is the lack of visuoperceptual function measures. The first two versions of the UDS did not comprehensively collect data on visuospatial skills, hence due to limited data availability (from the implementation of third UDS version) we did not explore the respective presymptomatic trajectories.

### Electronic supplementary material

Below is the link to the electronic supplementary material.

Supplementary Material 1

Supplementary Material 2

## Data Availability

No datasets were generated or analysed during the current study.
