# Supplementary material for: Cognitive trajectories preluding the onset of different dementia entities: a descriptive longitudinal study using the NACC database
Source: Aging Clin Exp Res. 2024 May 23;36(1):119. doi: 10.1007/s40520-024-02769-9 (PMC11116253; doi:10.1007/s40520-024-02769-9)
Supplement: Supplementary file 1 — Supplementary Material 1 [file 40520_2024_2769_MOESM1_ESM.docx]

**Supplementary Figure 1** Flowchart of participants’ selection.

**47,165** total participants

**14,784** participants without follow-up

**3,398 cognitively unimpaired participants with a minimum monitoring of 6 visits (which corresponds to at least 4.5 years of follow-up)**

**15,516** participants were excluded due to developing dementia associated with other principal aetiologies or progressing to cognitive impairment – not dementia or having fewer than 6 total assessments

**18,914** subjects without dementia at baseline and with at least one follow-up assessment that did not progress to AD, LBD, FTLD, VD

**247 converted to Lewy body dementia (LBD)**

**3,343 progressed to Alzheimer’s disease dementia (AD)**

**155 were diagnosed with vascular dementia (VD)**

**22,767** adults without dementia at baseline and with at least one follow-up assessment

**9,614** participants with dementia at baseline

**32,381** individuals at baseline with at least one follow-up assessment

**Supplementary Figure 2** Radar plots illustrating relative (to healthy controls) cognitive deficits per time point: 10 years prior to dementia onset, 3 years prior to dementia onset and at the time of the formal diagnosis. The external regular pentagon represents the performance of those without cognitive impairment throughout the follow-up. Centripetally and respectively, the remaining pentagons correspond to 80%, 60%, 40% and 20% of the performance of the cognitively unimpaired sample. AD: Alzheimer’s disease dementia; LBD: Lewy body dementia; VD: vascular dementia; FTD: frontotemporal dementia.

**108 developed frontotemporal dementia (FTD)**
