# Supplementary material for: Cognitive trajectories preluding the onset of different dementia entities: a descriptive longitudinal study using the NACC database
Source: Aging Clin Exp Res. 2024 May 23;36(1):119. doi: 10.1007/s40520-024-02769-9 (PMC11116253; doi:10.1007/s40520-024-02769-9)
Supplement: Supplementary file 2 — Supplementary Material 2 [file 40520_2024_2769_MOESM2_ESM.docx]

**Supplementary Table 1** Participants’ demographics.

| **Parameter (at dementia onset)** | | **Alzheimer’s disease** | **Lewy body dementia** | **Frontotemporal dementia** | **Vascular dementia** | **Cognitively unimpaired individuals with at least 6 years of follow-up** |
| --- | --- | --- | --- | --- | --- | --- |
| **Age in years at the time of dementia onset** | | 80.4 ±8.2 | 76.0 ±7.2 | 71.9 ±6.5 | 84.6 ±7.3 | 81.1 ±7.9 |
| **Education in years of formal schooling** | | 15.5 ±3.3 | 16.1 ±3.5 | 15.8 ±2.8 | 15.0 ±3.8 | 16.1 ±3.1 |
| **Sex (male%)** | | 1518 (45.4) | 201 (81.4) | 55 (50.9) | 66 (42.6) | 1116 (32.8) |
| **Race (%)** | **Caucasian** | 2848 (85.5) | 231 (93.9) | 101 (93.5) | 120 (77.4) | 2761 (81.3) |
|  | **African American** | 342 (10.3) | 9 (3.7) | 2 (1.9) | 27 (17.4) | 531 (15.6) |
|  | **American Indian or Alaskan native** | 8 (0.2) | 1 (0.4) | 1 (0.9) | 1 (0.6) | 19 (0.6) |
|  | **native Hawaiian or Pacific islander** | 2 (0.1) | 0 (0.0) | 0 (0.0) | 0 (0.0) | 1 (0.0) |
|  | **Asian** | 87 (2.6) | 5 (2.0) | 4 (3.7) | 7 (4.5) | 74 (2.2) |
|  | **other** | 45 (1.4) | 0 (0.0) | 0 (0.0) | 0 (0.0) | 8 (0.2) |

**Supplementary Table 2** Participants analysed per annual assessment - per dementia group (the minimum number of those with available data among the six cognitive assessments is provided).

| **Dementia at follow-up** | **-10 years** | **-9 years** | **-8 years** | **-7 years** | **-6 years** | **-5 years** | **-4 years** | **-3 years** | **-2 years** | **-1 years** | **Year of onset** |
| --- | --- | --- | --- | --- | --- | --- | --- | --- | --- | --- | --- |
| **AD**  **(mean)** | 229 | 303 | 385 | 495 | 620 | 789 | 1,001 | 1,287 | 1,688 | 2,260 | 2,273 |
| **LBD**  **(mean)** | 10 | 17 | 21 | 23 | 28 | 38 | 54 | 82 | 122 | 150 | 135 |
| **FTD**  **(mean)** | NA | NA | NA | NA | NA | NA | NA | 14 | 37 | 70 | 64 |
| **VD**  **(mean)** | 17 | 21 | 28 | 34 | 41 | 57 | 68 | 80 | 89 | 92 | 87 |
| **Controls (mean)** | 1,109 | 1,437 | 1,765 | 2,100 | 2,506 | 2,747 | 2,766 | 2,697 | 2,197 | 1,610 | 2,230 |

AD: Alzheimer’s disease dementia; LBD: Lewy body dementia; VD: vascular dementia; FTD: frontotemporal dementia; Controls: cognitively unimpaired individuals with at least 6 years of follow-up; NA: non-applicable – fewer than 10.

**Supplementary Table 3** Adjusted episodic memory scores (Logical Memory Test Sory -A / Delayed Recall) prior to dementia onset, throughout the 10-year preclinical follow-up.

| **Dementia at follow-up** | **-10 years** | **-9 years** | **-8 years** | **-7 years** | **-6 years** | **-5 years** | **-4 years** | **-3 years** | **-2 years** | **-1 years** | **Year of onset** |
| --- | --- | --- | --- | --- | --- | --- | --- | --- | --- | --- | --- |
| **Upper CI** | 14,02 | 14,04 | 13,69 | 13,35 | 13,03 | 10,57 | 10,53 | 7,59 | 7,28 | 5,53 | 4,27 |
| **AD**  **(mean)** | 12,36 | 12,50 | 12,23 | 11,88 | 11,56 | 9,87 | 9,04 | 7,02 | 5,77 | 4,61 | 3,42 |
| **Lower CI** | 10,71 | 10,97 | 10,77 | 10,40 | 10,10 | 9,17 | 7,55 | 6,44 | 4,25 | 3,70 | 2,56 |
| **Upper CI** | 14,65 | 13,89 | 12,96 | 13,20 | 13,17 | 11,96 | 11,61 | 9,73 | 10,03 | 8,58 | 7,31 |
| **LBD**  **(mean)** | 11,76 | 11,55 | 10,83 | 11,07 | 11,12 | 9,49 | 9,76 | 8,68 | 8,34 | 7,46 | 6,29 |
| **Lower CI** | 8,87 | 9,20 | 8,70 | 8,93 | 9,08 | 9,02 | 7,91 | 7,62 | 6,65 | 6,35 | 5,26 |
| **Upper CI** |  |  |  |  |  |  |  | 10,03 | 8,68 | 7,00 | 5,62 |
| **FTD**  **(mean)** |  |  |  |  |  |  |  | 7,96 | 6,65 | 5,64 | 4,35 |
| **Lower CI** |  |  |  |  |  |  |  | 5,89 | 4,62 | 4,29 | 3,08 |
| **Upper CI** | 14,89 | 13,48 | 13,64 | 12,87 | 13,23 | 11,84 | 12,77 | 10,48 | 11,36 | 9,64 | 7,88 |
| **VD**  **(mean)** | 12,45 | 11,28 | 11,65 | 10,92 | 11,36 | 10,58 | 11,01 | 9,44 | 9,63 | 8,43 | 6,77 |
| **Lower CI** | 10,01 | 9,08 | 9,67 | 8,97 | 9,49 | 9,33 | 9,25 | 8,41 | 7,90 | 7,22 | 5,66 |
| **Upper CI** | 15,53 | 15,73 | 16,09 | 16,01 | 16,11 | 14,66 | 15,53 | 14,21 | 15,52 | 15,11 | 15,11 |
| **Controls (mean)** | 13,92 | 14,24 | 14,67 | 14,57 | 14,68 | 14,00 | 14,06 | 13,65 | 14,01 | 14,19 | 14,25 |
| **Lower CI** | 12,32 | 12,75 | 13,25 | 13,12 | 13,24 | 13,34 | 12,59 | 13,09 | 12,51 | 13,27 | 13,40 |

Cognitive scores are adjusted for age, sex, race, education and time from dementia onset; AD: Alzheimer’s disease dementia; LBD: Lewy body dementia; VD: vascular dementia; FTLD: frontotemporal lobal degeneration dementia; Controls: cognitively unimpaired individuals with at least 6 years of follow-up; CI: confidence interval.

**Participants with AD** exhibited lower episodic memory scores **A)** compared to healthy controls from year -10 [-1.56 (-2.33, -0.79), p< .001] to dementia onset [-10.83 (-11.15, -10.52), p< .001]; **B)** compared to those with VD from year -4 [-1.97 (-3.42, -0.53), p= .001] to dementia onset [-3.35 (-4.41, -2.29), p< .001]; **C)** compared to those with LBD from year -3 [-1.66 (-2.99, -0.33), p= 0.005] to dementia onset [-2.87 (-3.72, -2.02), p< .001]; **D)** and similar scores to those with FTLD throughout the 3-year follow-up. **Participants with FTLD** performed **A)** worse than healthy controls from year -3 [-5.69 (-8.56, -2.83), p< .001] to dementia onset [-9.90 (-11.27, -8.53), p< .001]; **B)** worse than those with VD from year -2 [-2.98 (-5.30, -0.67), p= .003] to dementia onset [-2.42 (-4.13, -0.72), p= .001]; **C)** worse than those with LBD from year -1 [-1.82 (-3.51, -0.14), p= .024] to dementia onset [-1.94 (-3.51, -0.37), p= .005]; **D)** and similar to those with AD throughout the 3-year follow-up. **Participants with LBD** performed **A)** worse than healthy controls from year -9 [-2.69 (-5.15, -0.23), p= .024] to dementia onset [-7.96 (-8.82, -7.10), p< .001]; **B)** better than those with AD and **C)** FTLD (as described above) **D)** and similar to those with VD throughout the 10-year follow-up. **Participants with VD** performed **A)** worse than healthy controls from year -9 [-2.96 (-5.17, -0.75), p= .003] to dementia onset [-7.48 (-8.54, -6.42), p< .001]; **B)** better than those with AD and **C)** FTLD (as described above) **D)** and similar to those with LBD throughout the 10-year follow-up.

**Supplementary Table 4** Adjusted category fluency scores (sum of animal & vegetable lists) prior to dementia onset, throughout the 10-year preclinical follow-up.

| **Dementia at follow-up** | **-10 years** | **-9 years** | **-8 years** | **-7 years** | **-6 years** | **-5 years** | **-4 years** | **-3 years** | **-2 years** | **-1 years** | **Year of onset** |
| --- | --- | --- | --- | --- | --- | --- | --- | --- | --- | --- | --- |
| **Upper CI** | 37,73 | 34,35 | 34,50 | 33,07 | 32,10 | 27,97 | 27,27 | 25,73 | 24,71 | 22,99 | 19,87 |
| **AD**  **(mean)** | 34,51 | 31,24 | 31,51 | 30,21 | 29,34 | 26,71 | 24,61 | 24,71 | 22,09 | 21,38 | 18,25 |
| **Lower CI** | 31,30 | 28,13 | 28,52 | 27,35 | 26,58 | 25,44 | 21,95 | 23,70 | 19,48 | 19,77 | 16,63 |
| **Upper CI** | 35,05 | 34,83 | 33,28 | 32,20 | 31,73 | 27,28 | 28,03 | 25,57 | 25,01 | 23,54 | 19,63 |
| **LBD**  **(mean)** | 29,44 | 30,08 | 28,93 | 28,05 | 27,88 | 24,59 | 24,73 | 23,71 | 22,10 | 21,59 | 17,69 |
| **Lower CI** | 23,83 | 25,33 | 24,57 | 23,90 | 24,02 | 21,90 | 21,43 | 21,86 | 19,19 | 19,64 | 15,75 |
| **Upper CI** |  |  |  |  |  |  |  | 21,24 | 17,55 | 16,03 | 13,73 |
| **FTD**  **(mean)** |  |  |  |  |  |  |  | 17,48 | 14,05 | 13,67 | 11,31 |
| **Lower CI** |  |  |  |  |  |  |  | 13,72 | 10,55 | 11,31 | 8,88 |
| **Upper CI** | 36,64 | 35,46 | 34,27 | 35,27 | 33,39 | 29,18 | 27,43 | 26,33 | 25,42 | 23,99 | 20,63 |
| **VD**  **(mean)** | 31,99 | 30,99 | 30,21 | 31,46 | 29,87 | 26,93 | 24,29 | 24,50 | 22,42 | 21,87 | 18,52 |
| **Lower CI** | 27,33 | 26,53 | 26,16 | 27,66 | 26,34 | 24,67 | 21,15 | 22,67 | 19,42 | 19,74 | 16,42 |
| **Upper CI** | 40,47 | 37,90 | 38,30 | 37,38 | 36,74 | 33,86 | 34,31 | 33,50 | 33,95 | 33,30 | 33,26 |
| **Controls (mean)** | 37,35 | 34,88 | 35,39 | 34,58 | 34,03 | 32,66 | 31,67 | 32,51 | 31,34 | 31,69 | 31,64 |
| **Lower CI** | 34,24 | 31,86 | 32,48 | 31,77 | 31,33 | 31,46 | 29,04 | 31,53 | 28,73 | 30,08 | 30,02 |

Cognitive scores are adjusted for age, sex, race, education and time from dementia onset; AD: Alzheimer’s disease dementia; LBD: Lewy body dementia; VD: vascular dementia; FTLD: frontotemporal lobal degeneration dementia; Controls: cognitively unimpaired individuals with at least 6 years of follow-up; CI: confidence interval.

**Participants with FTLD** performed **A)** worse than healthy controls from year -3 [-15.03 (-20.24, -9.82), p< .001] to dementia onset [-20.34 (-22.96, -17.71), p< .001]; **B)** worse than those with VD from year -3 [-7.02 (-12.70, -1.33), p= .005] to dementia onset [-7.22 (-10.48, -3.96), p< .001]; **C)** worse than those with LBD from year -3 [-6.23 (-11.88, -0.58), p= .020] to dementia onset [-6.38 (-9.39, -3.38), p< .001]; **D)** worse than those with AD from year -3 [-7.23 (-12.46, -2.00), p= .001] to dementia onset [-6.94 (-9.56, -4.32), p< .001]. **Participants with AD** exhibited **A)** lower verbal fluency scores compared to healthy controls from year -10 [-2.84 (-4.33, -1.35), p< .001] to dementia onset [-13.39 (-13.98, -12.80), p< .001]; **B)** higher scores compared to those with FTLD (as described above); **C)** similar scores to those with VD and **D)** LBD throughout the 10-year follow-up. **Participants with VD** exhibited **A)** lower verbal fluency scores compared to healthy controls from year -10 [-5.37 (-10.11, -0.63), p= .017] to dementia onset [-13.12 (-15.12, -11.11), p< .001]; **B)** higher scores compared to those with FTLD (as described above); **C)** similar scores to those with AD and **D)** LBD throughout the 10-year follow-up. **Participants with LBD** exhibited **A)** lower verbal fluency scores compared to healthy controls from year -10 [-7.91 (-14.25, -1.58), p= .006] to dementia onset [-13.95 (-15.58, -12.33), p< .001]; **B)** higher scores compared to those with FTLD (as described above); **C)** similar scores to those with VD and **D)** AD throughout the 10-year follow-up.

**Supplementary Table 5** Adjusted confrontation naming scores (Boston Naming Test – 30) prior to dementia onset, throughout the 10-year preclinical follow-up.

| **Dementia at follow-up** | **-10 years** | **-9 years** | **-8 years** | **-7 years** | **-6 years** | **-5 years** | **-4 years** | **-3 years** | **-2 years** | **-1 years** | **Year of onset** |
| --- | --- | --- | --- | --- | --- | --- | --- | --- | --- | --- | --- |
| **Upper CI** | 27,25 | 27,08 | 26,65 | 26,48 | 26,36 | 24,84 | 25,53 | 24,07 | 23,52 | 21,81 | 21,12 |
| **AD**  **(mean)** | 26,18 | 26,08 | 25,66 | 25,48 | 25,37 | 24,38 | 24,51 | 23,65 | 23,02 | 20,75 | 20,16 |
| **Lower CI** | 25,11 | 25,09 | 24,68 | 24,48 | 24,39 | 23,92 | 23,48 | 23,22 | 22,51 | 19,69 | 19,21 |
| **Upper CI** | 27,67 | 27,60 | 27,16 | 26,70 | 26,46 | 25,39 | 25,80 | 24,61 | 24,61 | 22,89 | 22,37 |
| **LBD**  **(mean)** | 25,80 | 26,08 | 25,73 | 25,25 | 25,08 | 24,44 | 24,52 | 23,84 | 23,83 | 21,67 | 21,21 |
| **Lower CI** | 23,93 | 24,56 | 24,29 | 23,80 | 23,71 | 23,48 | 23,25 | 23,07 | 23,05 | 20,44 | 20,06 |
| **Upper CI** |  |  |  |  |  |  |  | 22,88 | 20,03 | 16,46 | 13,26 |
| **FTD**  **(mean)** |  |  |  |  |  |  |  | 21,17 | 18,80 | 15,02 | 11,84 |
| **Lower CI** |  |  |  |  |  |  |  | 19,47 | 17,58 | 13,59 | 10,43 |
| **Upper CI** | 26,90 | 26,99 | 26,86 | 26,38 | 26,27 | 24,26 | 25,30 | 23,76 | 24,09 | 22,36 | 21,90 |
| **VD**  **(mean)** | 25,33 | 25,57 | 25,52 | 25,05 | 25,02 | 23,45 | 24,09 | 23,00 | 23,25 | 21,05 | 20,65 |
| **Lower CI** | 23,75 | 24,14 | 24,18 | 23,71 | 23,76 | 22,64 | 22,88 | 22,23 | 22,40 | 19,74 | 19,41 |
| **Upper CI** | 27,78 | 27,97 | 27,70 | 27,70 | 27,59 | 26,33 | 27,48 | 26,60 | 26,73 | 25,81 | 26,59 |
| **Controls (mean)** | 26,75 | 27,00 | 26,74 | 26,72 | 26,62 | 25,90 | 26,47 | 26,19 | 26,23 | 24,74 | 25,63 |
| **Lower CI** | 25,71 | 26,04 | 25,78 | 25,74 | 25,66 | 25,47 | 25,45 | 25,78 | 25,73 | 23,66 | 24,67 |

Cognitive scores are adjusted for age, sex, race, education and time from dementia onset; AD: Alzheimer’s disease dementia; LBD: Lewy body dementia; VD: vascular dementia; FTLD: frontotemporal lobal degeneration dementia; Controls: cognitively unimpaired individuals with at least 6 years of follow-up; CI: confidence interval.

**Participants with FTLD** performed **A)** worse than healthy controls from year -3 [-5.02 (-7.39, -2.65), p< .001] to dementia onset [-13.79 (-15.30, -12.27), p< .001]; **B)** worse than those with VD from year -2 [-4.44 (-6.36, -2.53), p< .001] to dementia onset [-8.81 (-10.71, -6.91), p< .001]; **C)** worse than those with LBD from year -3 [-2.67 (-5.21, -0.13), p= .032] to dementia onset [-9.37 (-11.12, -7.62), p< .001]; **D)** worse than those with AD from year -3 [-2.47 (-4.85, -0.09), p= .035] to dementia onset [-8.32 (-9.83, -6.81), p< .001]. **Participants with AD** exhibited **A)** lower naming scores compared to healthy controls from year -10 [-0.57 (-1.06, -0.07), p< .016] to dementia onset [-5.47 (-5.84, -5.10), p< .001]; **B)** higher scores compared to those with FTLD (as described above); **C)** similar scores to those with VD and **D)** LBD [apart from the year of dementia onset when those with AD performed slightly worse than those with LBD: -1.05 (-2.01, -0.09), p= .022)]. **Participants with VD** exhibited **A)** lower verbal fluency scores compared to healthy controls from year -7 [-1.67 (-2.90, -0.44), p= .002] to dementia onset [-4.98 (-6.18, -3.78), p< .001]; **B)** higher scores compared to those with FTLD (as described above); **C)** similar scores to those with AD and **D)** LBD throughout the 10-year follow-up. **Participants with LBD** exhibited **A)** lower verbal fluency scores compared to healthy controls from year -7 [-1.47 (-2.93, -0.01), p= .048] to dementia onset [-4.42 (-5.40, -3.44), p< .001]; **B)** higher scores compared to those with FTLD (as described above); **C)** similar scores to those with VD and **D)** AD (only at the year of dementia onset those with AD performed slightly worse than those with LBD).

**Supplementary Table 6** Adjusted executive function scores (Trail Making Test -B) prior to dementia onset, throughout the 10-year preclinical follow-up.

| **Dementia at follow-up** | **-10 years** | **-9 years** | **-8 years** | **-7 years** | **-6 years** | **-5 years** | **-4 years** | **-3 years** | **-2 years** | **-1 years** | **Year of onset** |
| --- | --- | --- | --- | --- | --- | --- | --- | --- | --- | --- | --- |
| **Upper CI** | 112,95 | 120,97 | 126,02 | 127,79 | 139,85 | 136,52 | 151,89 | 147,97 | 172,63 | 193,07 | 224,63 |
| **AD**  **(mean)** | 98,47 | 105,51 | 111,90 | 112,41 | 125,00 | 128,63 | 135,46 | 140,47 | 162,89 | 175,44 | 207,13 |
| **Lower CI** | 83,99 | 90,06 | 97,79 | 97,03 | 110,16 | 120,73 | 119,04 | 132,98 | 153,16 | 157,81 | 189,64 |
| **Upper CI** | 144,27 | 133,58 | 138,61 | 156,06 | 161,63 | 171,52 | 185,24 | 205,15 | 236,03 | 249,21 | 279,87 |
| **LBD**  **(mean)** | 118,99 | 110,02 | 117,79 | 133,30 | 140,75 | 155,48 | 164,79 | 191,98 | 221,92 | 228,61 | 258,92 |
| **Lower CI** | 93,71 | 86,45 | 96,96 | 110,54 | 119,86 | 139,43 | 144,35 | 178,81 | 207,81 | 208,02 | 237,98 |
| **Upper CI** |  |  |  |  |  |  |  | 175,70 | 199,38 | 213,78 | 225,09 |
| **FTD**  **(mean)** |  |  |  |  |  |  |  | 150,48 | 178,86 | 190,08 | 200,79 |
| **Lower CI** |  |  |  |  |  |  |  | 125,26 | 158,34 | 166,37 | 176,49 |
| **Upper CI** | 120,43 | 130,11 | 135,65 | 128,47 | 145,29 | 161,32 | 168,83 | 177,85 | 194,63 | 230,10 | 269,19 |
| **VD**  **(mean)** | 99,44 | 107,97 | 116,33 | 108,06 | 126,18 | 147,64 | 149,22 | 164,77 | 179,21 | 208,00 | 246,73 |
| **Lower CI** | 78,46 | 85,83 | 97,01 | 87,66 | 107,08 | 133,97 | 129,61 | 151,70 | 163,79 | 185,89 | 224,28 |
| **Upper CI** | 100,39 | 107,26 | 110,04 | 106,92 | 115,67 | 112,19 | 119,48 | 110,31 | 122,42 | 126,10 | 135,06 |
| **Controls (mean)** | 86,37 | 92,28 | 96,31 | 91,88 | 101,12 | 104,71 | 103,24 | 103,07 | 112,80 | 108,31 | 117,56 |
| **Lower CI** | 72,35 | 77,30 | 82,58 | 76,83 | 86,56 | 97,23 | 86,99 | 95,82 | 103,17 | 90,51 | 100,06 |

Cognitive scores are adjusted for age, sex, race, education and time from dementia onset; AD: Alzheimer’s disease dementia; LBD: Lewy body dementia; VD: vascular dementia; FTLD: frontotemporal lobal degeneration dementia; Controls: cognitively unimpaired individuals with at least 6 years of follow-up; CI: confidence interval.

**Participants with LBD** performed **A)** worse than healthy controls from year -10 [32.6 (4.1, 61.2), p= .015] to dementia onset [141.4 (124.0, 158.7), p< .001] (executive function scores were similar only at year -9); **B)** worse than those with AD from year -5 [26.9 (7.1, 46.6), p= .002] to dementia onset [51.8 (34.6, 69.0), p< .001]; **C)** worse than those with FTLD from year -3 [41.5 (3.6, 79.4), p= .021] to dementia onset [58.1 (28.9, 87.4), p< .001]; **D)** and similar to those with VD [temporary differences were observed at years -3 (27.2, (4.6, 49,8)) and -2 (42.7 (19.4, 66.1))]. **Participants with VD** exhibited worse executive function scores **A)** compared to healthy controls from year -8 [20.0 (1.5, 38.6), p= .026] to dementia onset [129.2 (108.1, 150.2), p< .001] (executive function scores were similar at year -7); **B)** compared to those with AD from year -5 [26.0 (9.8, 42.3), p< .001] to dementia onset [39.6 (18.6, 60.6), p< .001] (executive function scores were similar at years -4 and -2); **C)** compared to those with FTLD only at the time of dementia onset [45.9 (14.0, 77.9), p= .001]; **D)** and had similar executive function scores to those with LBD [temporary differences were observed at years -3 and -2]. **Participants with FTLD** performed **A)** worse than healthy controls from year -3 [47.4 (12.7, 82.1), p= .001] to dementia onset [83.2 (58.6, 107.9), p< .001]; **B)** better than those with LBD and **C)** VD (as described above); D) and similar to those with AD throughout the 3-year follow-up. **Participants with AD** exhibited **A)** worse executive function scores compared to healthy controls from year -10 [12.1 (5.4, 18.8), p< .001] to dementia onset [89.6 (83.7, 95.5), p< .001]; **B)** better scores compared to those with LBD and **C)** VD (as described above); **D)** and similar scores to those with FTLD throughout the 3-year follow-up.

**Supplementary Table 7** Adjusted processing speed – attention scores (Trail Making Test -A) prior to dementia onset, throughout the 10-year preclinical follow-up.

| **Dementia at follow-up** | **-10 years** | **-9 years** | **-8 years** | **-7 years** | **-6 years** | **-5 years** | **-4 years** | **-3 years** | **-2 years** | **-1 years** | **Year of onset** |
| --- | --- | --- | --- | --- | --- | --- | --- | --- | --- | --- | --- |
| **Upper CI** | 37,08 | 40,10 | 43,47 | 42,73 | 42,52 | 43,57 | 48,27 | 49,30 | 54,24 | 61,68 | 68,13 |
| **AD**  **(mean)** | 32,46 | 35,42 | 39,00 | 38,16 | 38,20 | 41,23 | 43,26 | 47,22 | 51,73 | 56,31 | 62,69 |
| **Lower CI** | 27,85 | 30,73 | 34,52 | 33,58 | 33,88 | 38,90 | 38,26 | 45,13 | 49,22 | 50,94 | 57,25 |
| **Upper CI** | 45,50 | 44,43 | 49,71 | 52,00 | 49,29 | 53,93 | 55,90 | 60,49 | 68,98 | 81,50 | 97,01 |
| **LBD**  **(mean)** | 37,44 | 37,28 | 43,19 | 45,37 | 43,27 | 49,17 | 49,70 | 56,65 | 65,13 | 75,31 | 90,45 |
| **Lower CI** | 29,39 | 30,13 | 36,67 | 38,75 | 37,24 | 44,40 | 43,50 | 52,81 | 61,28 | 69,11 | 83,89 |
| **Upper CI** |  |  |  |  |  |  |  | 44,13 | 56,78 | 65,24 | 69,31 |
| **FTD**  **(mean)** |  |  |  |  |  |  |  | 51,59 | 62,47 | 72,37 | 77,10 |
| **Lower CI** |  |  |  |  |  |  |  | 36,67 | 51,09 | 58,10 | 61,52 |
| **Upper CI** | 43,10 | 46,60 | 50,14 | 48,45 | 47,49 | 51,76 | 55,64 | 59,18 | 59,49 | 69,58 | 88,43 |
| **VD**  **(mean)** | 36,42 | 39,88 | 44,06 | 42,41 | 41,92 | 47,66 | 49,72 | 55,38 | 55,27 | 62,86 | 81,26 |
| **Lower CI** | 29,73 | 33,17 | 37,99 | 36,37 | 36,36 | 43,55 | 43,79 | 51,59 | 51,05 | 56,13 | 74,09 |
| **Upper CI** | 34,75 | 36,78 | 40,37 | 37,56 | 38,79 | 38,21 | 40,60 | 39,74 | 43,01 | 47,45 | 46,75 |
| **Controls (mean)** | 30,28 | 32,24 | 36,02 | 33,08 | 34,56 | 35,99 | 35,65 | 37,72 | 40,52 | 42,02 | 41,28 |
| **Lower CI** | 25,81 | 27,69 | 31,66 | 28,61 | 30,32 | 33,78 | 30,70 | 35,71 | 38,03 | 36,59 | 35,81 |

Cognitive scores are adjusted for age, sex, race, education and time from dementia onset; AD: Alzheimer’s disease dementia; LBD: Lewy body dementia; VD: vascular dementia; FTLD: frontotemporal lobal degeneration dementia; Controls: cognitively unimpaired individuals with at least 6 years of follow-up; CI: confidence interval.

**Participants with LBD** performed **A)** worse than healthy controls from year -8 [7.2 (0.5, 13.8), p= .026] to dementia onset [49.1 (43.6, 54.8), p< .001]; **B)** worse than those with AD from year -5 [7.9 (2.1, 13.8), p= .002] to dementia onset [27.8 (22.3, 33.3), p< .001]; **C)** worse than those with FTLD from year -3 [16.5 (5.2, 27.8), p< .001] to dementia onset [21.1 (11.6, 30.7), p< .001] (processing speed scores were similar only at year -2); **D)** worse than those with VD from year -2 [9.9, (3.2, 16,5), p< .001] to dementia onset [(9.2 (0.5, 17.9), p= .029]. **Participants with VD** exhibited worse processing speed scores **A)** compared to healthy controls from year -9 [7.6 (0.9, 14.4), p= .017] to dementia onset [40.0 (33.0, 47.0), p< .001]; **B)** compared to those with AD from year -5 [6.4 (1.5, 11.3), p= .003] to dementia onset [18.6 (11.6, 25.6), p< .001] (processing speed scores were similar at year -2); **C)** compared to those with FTLD only at the time of dementia onset [12.0 (1.4, 22.5), p= .015]; **D)** and better scores than those with LBD (as described above). **Participants with FTLD** performed **A)** worse than healthy controls from year -2 [16.3 (8.7, 23.8), p< .001] to dementia onset [28.0 (19.9, 36.2), p< .001]; **B)** better than those with LBD and **C)** VD (as described above); **D)** and similar to those with AD [a temporary difference was observed at year -1 (8.9, (2.1, 15,7))]. **Participants with AD** exhibited **A)** worse processing speed scores compared to healthy controls from year -10 [2.2 (0.0, 4.3), p= .043] to dementia onset [21.4 (19.3, 23.5), p< .001]; **B)** better scores compared to those with LBD and **C)** VD (as described above); **D)** and similar scores to those with FTLD (a temporary difference was observed at year).

**Supplementary Table 8** Adjusted rates of cognitive decline by dementia diagnosis at follow-up

| **Cognitive domain** | **β coefficients for the 10-year follow-up prior to dementia onset** | | | | |
| --- | --- | --- | --- | --- | --- |
|  | **Alzheimer’s disease** | **Lewy body dementia** | **Frontotemporal dementia** | **Vascular dementia** | **Cognitively unimpaired** |
| **Episodic Memory**  **(Delayed Recall)** | -0.87 (-0.91, -0.83)  p< .001 | -0.44 (-0.60, -0.28)  p< .001 | Non-applicable | -0.48 (-0.60, -0.36)  p< .001 | Reference |
| **Category fluency (Vegetable & Animal lists)** | -1.13 (-1.20, -1.06)  p< .001 | -0.99 (-1.23, -0.76)  p< .001 | Non-applicable | -0.92 (-1.16, -0.68)  p< .001 | Reference |
| **Naming**  **(BNT-30)** | -0.47 (-0.50, -0.43)  p< .001 | -0.25 (-0.35, -0.16)  p< .001 | Non-applicable | -0.27 (-0.37, -0.17)  p< .001 | Reference |
| **Executive function (TMT-B)** | 8.5 (7.9, 9.0)  p< .001 | 16.0 (13.6, 18.4)  p< .001 | Non-applicable | 12.5 (10.5, 14.5)  p< .001 | Reference |
| **Processing speed-attention (TMT-A)** | 2.0 (1.8, 2.1)  p< .001 | 5.6 (4.7, 6.4)  p< .001 | Non-applicable | 3.2 (2.5, 4.0)  p< .001 | Reference |
| **Cognitive domain** | **β coefficients for the last four years prior to dementia onset** | | | | |
|  | **Alzheimer’s disease** | **Lewy body dementia** | **Frontotemporal dementia** | **Vascular dementia** | **Cognitively unimpaired** |
| **Episodic Memory**  **(Delayed Recall)** | -1.17 (-1.24, -1.10)  p< .001 | -0.79 (-1.05, -0.53)  p< .001 | -1.23 (-1.59, -0.88)  p< .001 | -0.98 (-1.25, -0.71)  p< .001 | Reference |
| **Category fluency (Vegetable & Animal lists)** | -1.71 (-1.82, -1.60)  p< .001 | -1.57 (-1.96, -1.17)  p< .001 | -2.45 (-3.17, -1.73)  p< .001 | -1.47 (-1.86, -1.09)  p< .001 | Reference |
| **Naming**  **(BNT-30)** | -0.84 (-0.90, -0.78)  p< .001 | -0.55 (-0.74, -0.35)  p< .001 | -2.73 (-3.55, -1.91)  p< .001 | -0.51 (-0.78. -0.25)  p< .001 | Reference |
| **Executive function (TMT-B)** | 16.4 (15.2, 17.6)  p< .001 | 21.5 (16.5, 26.5)  p< .001 | 16.0 (7.0, 24.9)  p< .001 | 23.9 (18.9, 29.0)  p< .001 | Reference |
| **Processing speed-attention (TMT-A)** | 3.7 (3.3, 4.1)  p< .001 | 10.4 (8.6, 12.2)  p< .001 | 6.0 (3.8, 8.3)  p< .001 | 7.5 (5.4, 9.6)  p< .001 | Reference |
